# Supplementary material for: Dietary L-Tryptophan consumption determines the number of colonic regulatory T cells and susceptibility to colitis via GPR15
Source: Nat Commun. 2023 Nov 14;14:7363. doi: 10.1038/s41467-023-43211-4 (PMC10645889; doi:10.1038/s41467-023-43211-4)
Supplement: Supplementary file 1 — Supplementary Information [file 41467_2023_43211_MOESM1_ESM.pdf]

Dietary L-Tryptophan consumption determines the number of colonic regulatory T cells and susceptibility to colitis via GPR15

Authors: Nguyen T. Van,[1,2,11] Karen Zhang,[1,2,11] Rachel M. Wigmore,[1,2] Anne I. Kennedy,[1,2] Carolina R. DaSilva,[1,2] Jialing Huang,[3,12] Manju Ambelil,[3] Jose H. Villagomez,[1,2] Gerald J. O'Connor,[1,2] Randy S. Longman,[4] Miao Cao,[5] Adam E. Snook,[1,2,5] Michael Platten,[6,7,8] Gerard Kasenty,[9] Luis J. Sigal,[1,2] George C. Prendergast,[3,10] and Sangwon V. Kim[1,2,*]

Affiliations:

[1] Department of Microbiology and Immunology, Sidney Kimmel Medical College, Thomas Jefferson University, Philadelphia, PA, USA.
[2] Sidney Kimmel Cancer Center, Jefferson Health, Philadelphia, PA, USA.
[3] Department of Pathology, Anatomy, & Cell Biology, Sidney Kimmel Medical College, Thomas Jefferson University, Philadelphia, PA, USA
[4] Jill Roberts Center for IBD, Weill Cornell Medicine, New York, NY, USA.
[5] Department of Pharmacology, Physiology, & Cancer Biology, Sidney Kimmel Medical College, Thomas Jefferson University, Philadelphia, PA, USA
[6] CCU Neuroimmunology and Brain Tumor Immunology, German Cancer Research Center, Heidelberg, Germany
[7] Department of Neurology, Medical Faculty Mannheim, MCTN, Heidelberg University, Heidelberg, Germany
[8] DKFZ Hector Cancer Institute at the University Medical Center Mannheim, Mannheim, Germany
[9] Department of Genetics and Development, Irving Medical Center, Columbia University, NY, USA
[10] Lankenau Institute of Medical Research, Wynnewood, PA, USA
[11] These authors contributed equally
[12] Present address: Anatomic Pathology, Geisinger Medical Center, Danville, PA, USA

*Corresponding author. Email: svkim@jefferson.edu

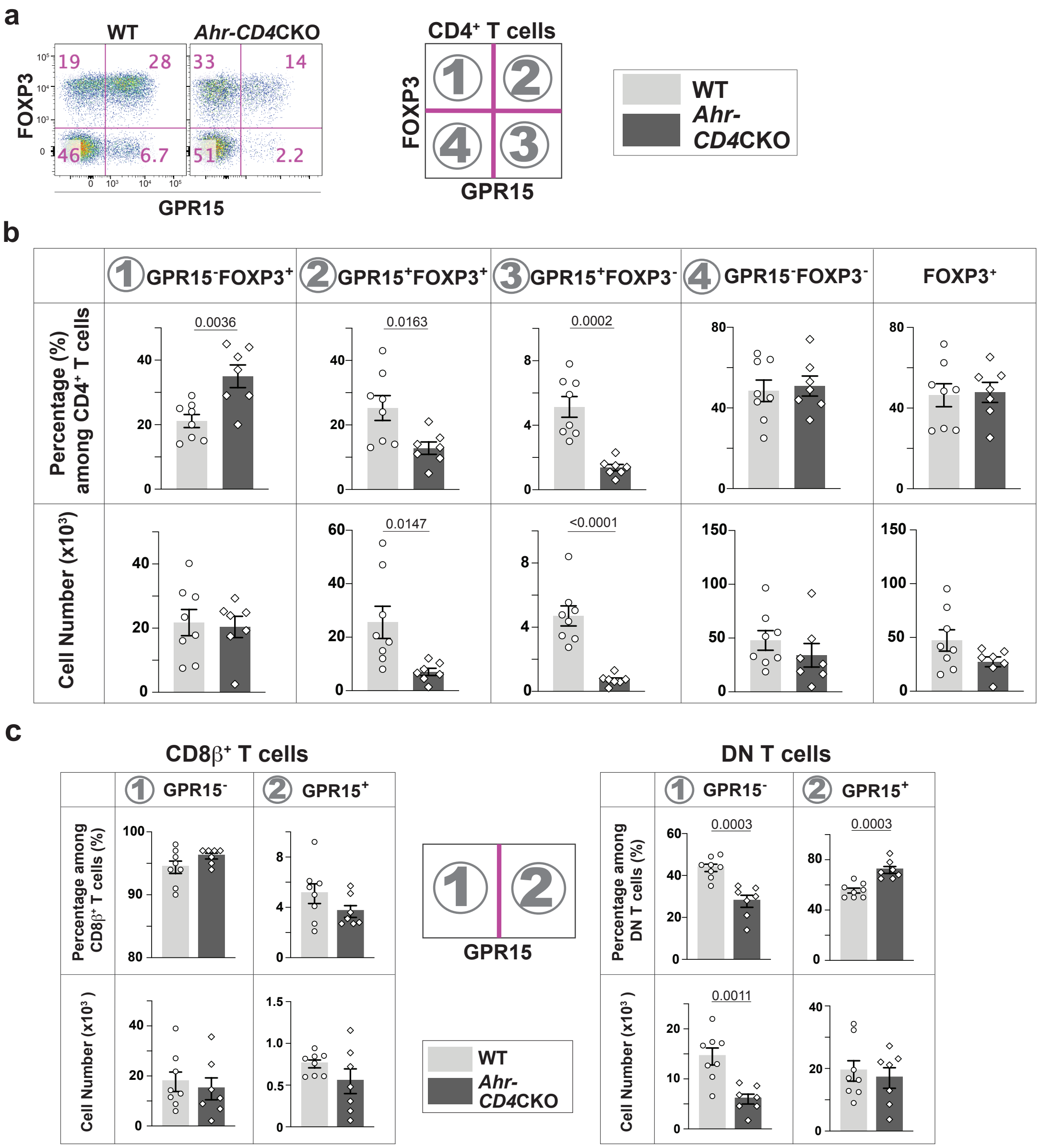

Supplementary Fig. 1 AhR is required for GPR15 expression in CD4$^+$ T cells, but not in CD8β$^+$ or CD4$^-$CD8β$^-$ (DN) T cells. **a-b**. GPR15 and FOXP3 protein expression in CD4$^+$ T cells in the large intestine lamina propria (LILP) at steady state. Wild-type mice (WT: *Ahr$^{(fl/fl)}$*, n=8) and T cell-specific *Ahr*-deficient mice (*Ahr-CD4*CKO: *CD4^{Cre}Ahr$^{(fl/fl)}$*, n=7) were used. Representative flow cytometry data, the percentage among CD4$^+$ T cells, and the total cell number of each population were shown. Representative of three independent experiments. **c.** CD8β$^+$ T cells and DN T cells in the LILP at a steady state were analyzed for GPR15 expression in the presence (*Ahr$^{(fl/fl)}$*; n=8) or absence of AhR (*CD4^{Cre}Ahr$^{(fl/fl)}$*; n=7). Representative of at least three independent experiments. 8-14-week-old mice in C57BL/6 background were used (**a-c**). Data are presented as mean values +/- SEM (**b-c**). Each data point represents the result from one mouse, and p values were calculated by two-sided student's t-test (**b-c**). Source data are provided as a Source Data file (**b-c**).

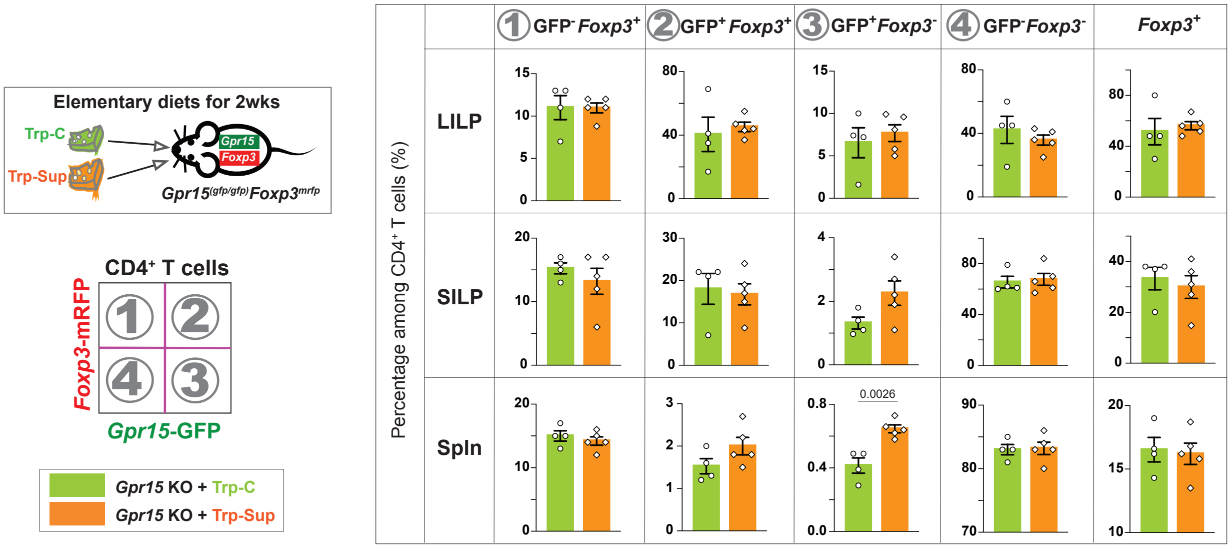

Supplementary Fig. 2 Dietary supplementation of L-Tryptophan increases GPR15^{+}CD4^{+} T cells and their subsequent migration to the LILP.

8-12-week-old *Gpr15$^{(gfp/gfp)}$Foxp3^{mrfp}* mice with TJU microbiota in C57BL/6 background were treated with Trp-C or Trp-Sup elementary diets for two weeks, and GPR15 wannabe (GFP^{+}) cells and their FOXP3 expression were examined (SILP: small intestine lamina propria; Spln: spleen). The number of mice used: 4 for Trp-C and 5 for Trp-Sup. Data are presented as mean values +/- SEM. Each data point represents the result from one mouse, and p values were calculated by two-sided student's t-test. Source data are provided as a Source Data file.

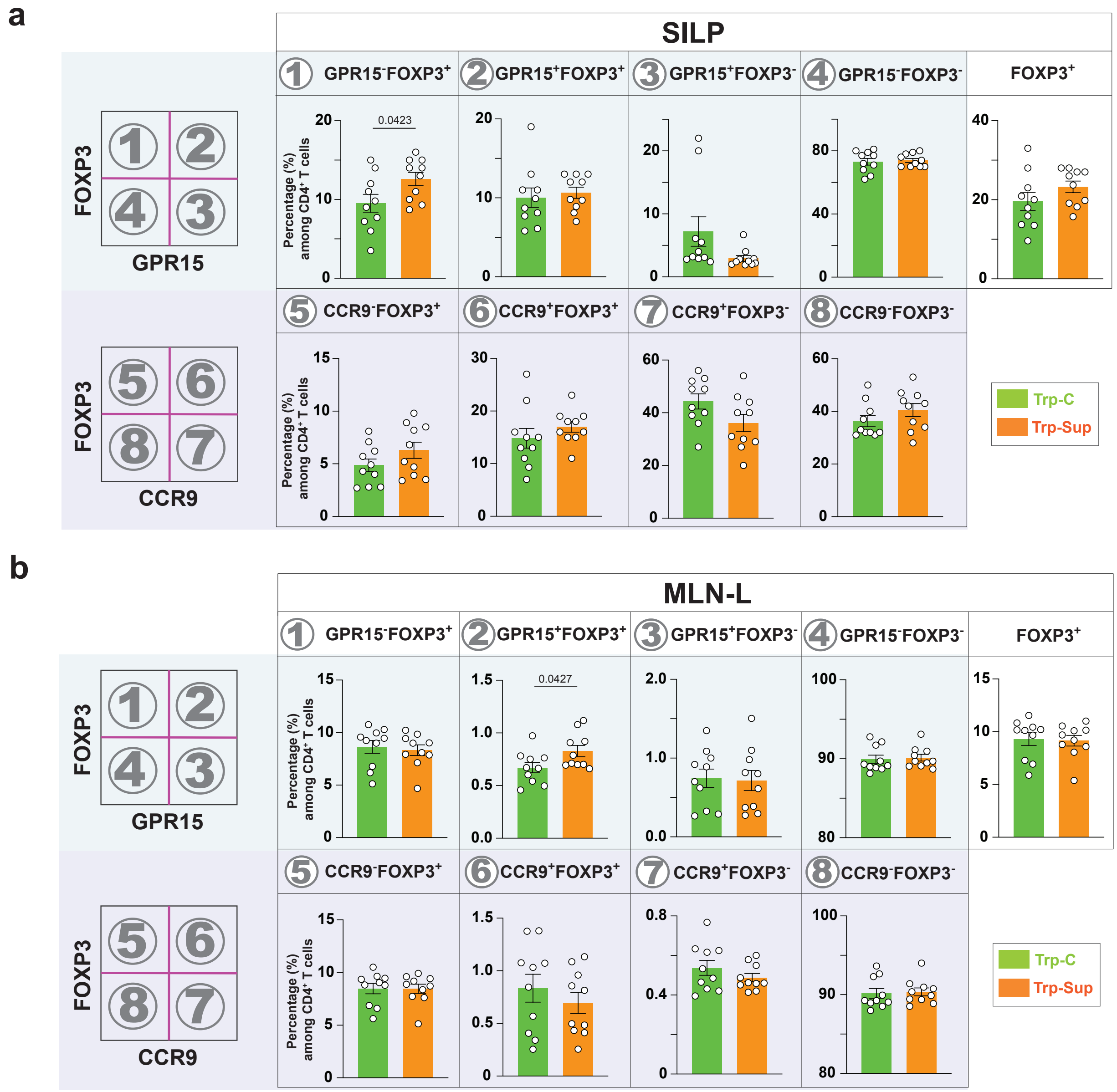

Supplementary Fig. 3 L-Trp supplementation increases GPR15^{+} Tregs in the lymph nodes draining the large intestine and does not induce CCR9 expression.

a-b. GPR15 (in the light blue shade), CCR9 (in the light purple shade), and FOXP3 expression in CD4^{+} T cells in the SILP (**a**) and MLNs-draining the proximal and mid-colon (MLN-L) (**b**). Trp-C (n=10), Trp-Sup (n=10). Combined results of two independent experiments (**a-b**). 8-12-week-old wild-type mice in C57BL/6 background were used (**a-b**). Data are presented as mean values +/- SEM (**a-b**). Each data point represents the result from one mouse, and p values were calculated by two-sided student's t-test(**a-b**). Source data are provided as a Source Data file (**a-b**).

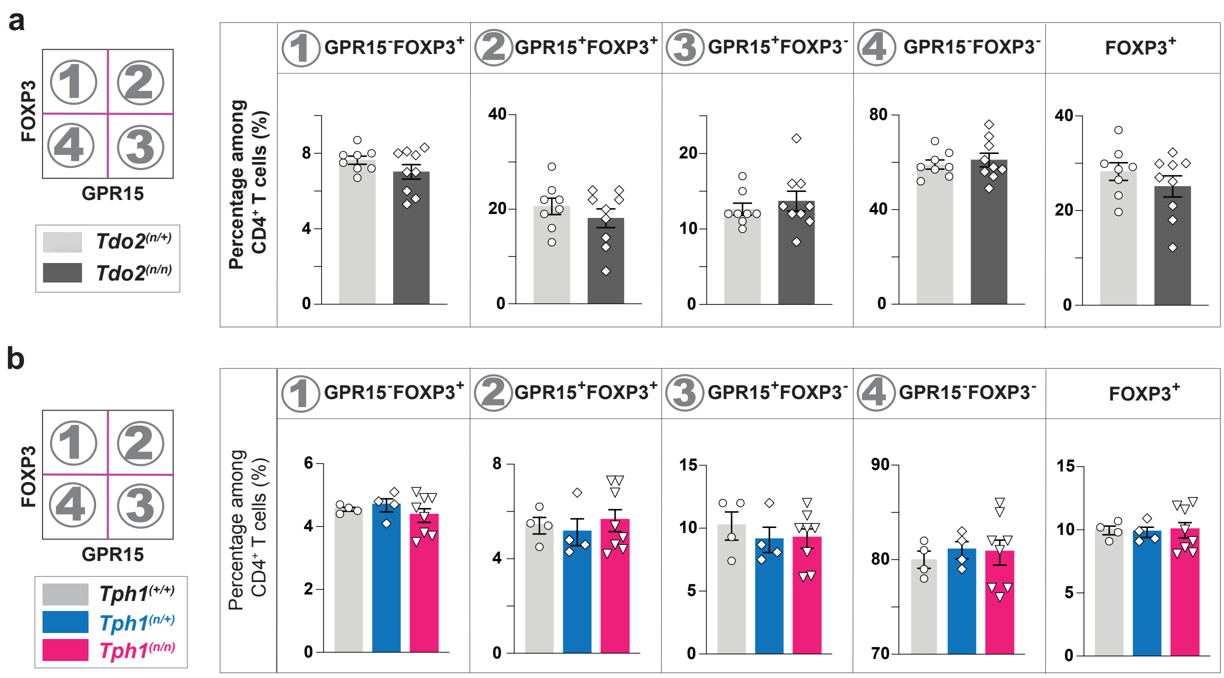

Supplementary Fig. 4 GPR15 expression in $CD4^+$ T cells in the large intestine is independent of host enzyme TDO and TPH1.

a. *Tdo2*$^{(n/+)}$ and *Tdo2*$^{(n/n)}$ mice were analyzed for GPR15 and FOXP3 expression among $CD4^+$ T cells in the LILP at a steady state. Number of mice used: 8 for *Tdo2*$^{(n/+)}$, and 9 for *Tdo2*$^{(n/n)}$. **b.** *Tph1*$^{(+/+)}$, *Tph1*$^{(n/+)}$, and *Tph1*$^{(n/n)}$ mice were analyzed for GPR15 and FOXP3 expression among $CD4^+$ T cells in the LILP at a steady state. Number of mice used: 4 for *Tph1*$^{(+/+)}$, 4 for *Tph1*$^{(n/+)}$ (n=4) and 8 for *Tph1*$^{(n/n)}$. Combined results of two independent experiments (**a-b**). 8-14-week-old mice in C57BL/6 background were used (**a-b**). Data are presented as mean values +/- SEM (**a-b**). Each data point represents the result from one mouse, and p values were calculated by two-sided student's t-test (**a-b**). Source data are provided as a Source Data file (**a-b**).

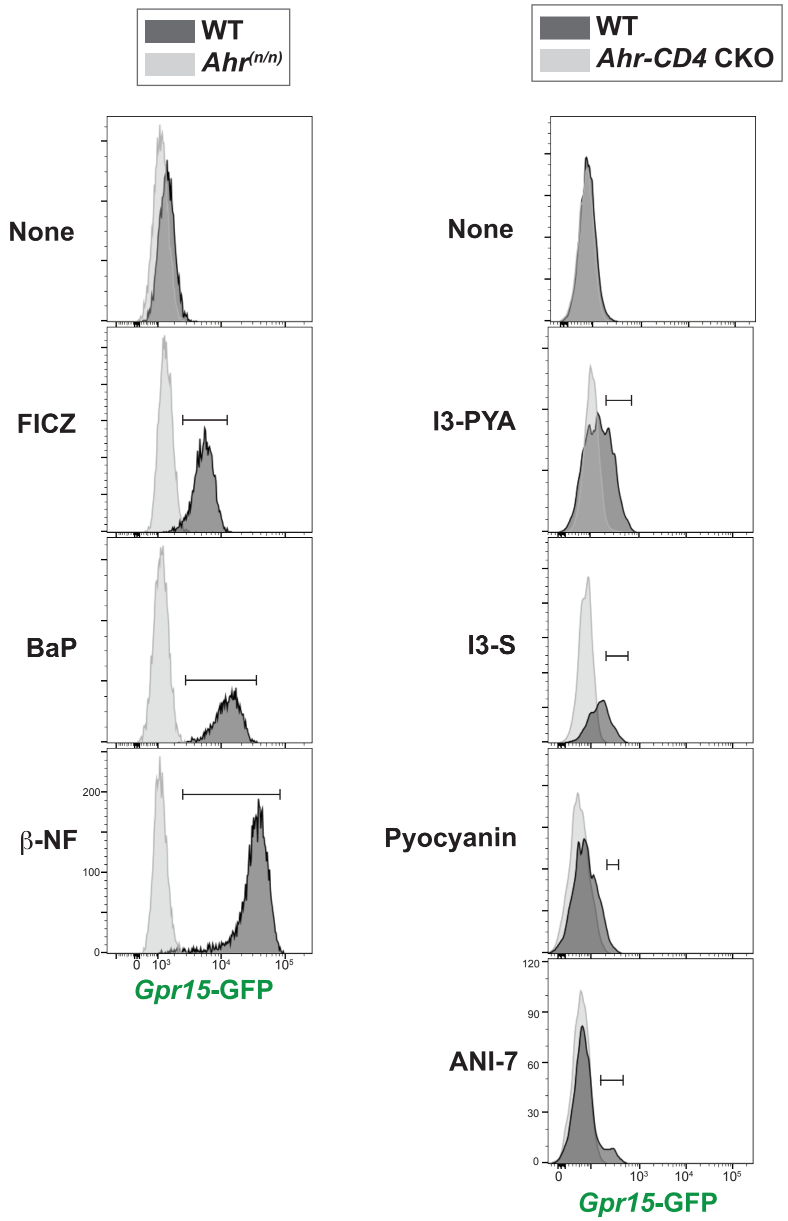

Supplementary Fig. 5 Seven selected chemical compounds induce *Gpr15*-GFP expression through AhR. CD4$^+$ naïve T cells (CD62L^{hi}CD44^{lo}mRFP$^-$CD25$^-$GFP$^-$CD4$^+$) from 8-14-week-old wild-type (*Ahr$^{(fl/fl)}$Gpr15$^{(gfp/+)}$Foxp3^{mrfp}*), or *Ahr* KO (*Ahr$^{(n/n}$Gpr15$^{(gfp/+)}$Foxp3^{mrfp}*), or *Ahr-CD4*CKO (*Cd4^{Cre}Ahr$^{(fl/fl)}$Gpr15$^{(gfp/+)}$Foxp3^{mrfp}*) mice in C57BL/6 background were stimulated with anti-CD3ε and anti-CD28 antibody for 2-3 days with or without hTGFβ1 (0-3 ng/ml) and the selected chemical compounds in Fig. 4A. The following concentration ranges of the compounds were used: I3-PYA (100-300 μM), I3-S (1-3 μM), Pyocyanin (300 nM-1 μM), FICZ (3-6 μM), ANI-7 (100-300 nM), BaP (100 nM-1 μM), b-NF (300 nM-1 μM). GFP signals dependent on AHR are labeled in the histograms (dark peaks: WT; gray peaks: KO or CKO).

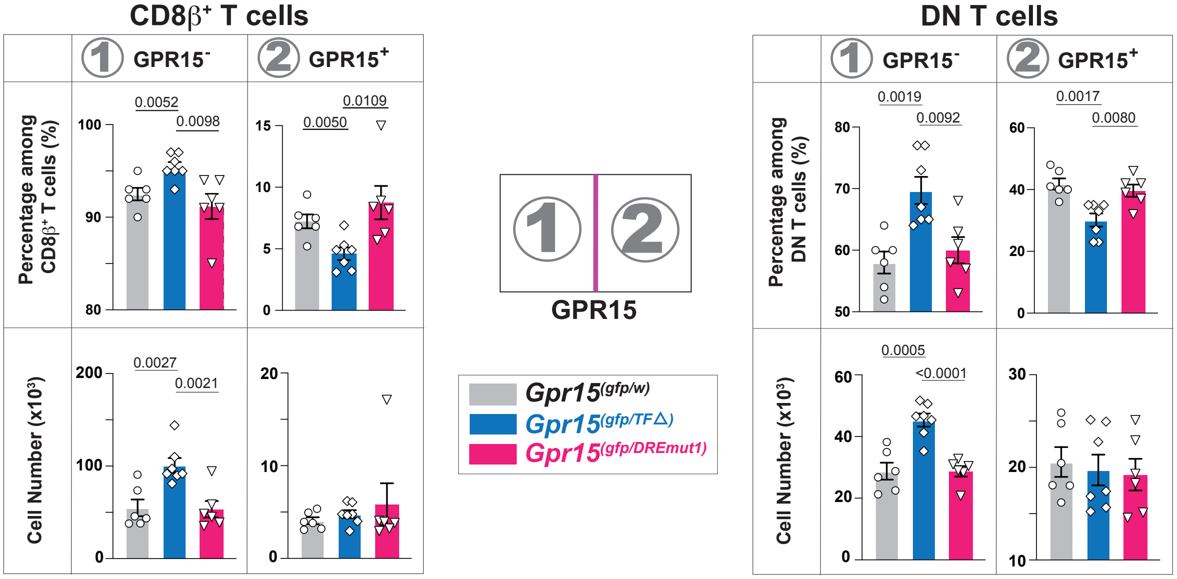

Supplementary Fig. 6 GPR15 expression in CD8β^{+} T cells and DN T cells in the LILP is affected by the binding of the transcriptional factors, other than AhR.
8-12-week-old *Gpr15*$^{(gfp/w)}$ (n=6), *Gpr15*$^{(gfp/\ TF\Delta)}$ (n=7), and *Gpr15*$^{(gfp/\ DREmut1)}$ (n=6) mice in C57BL/6 background. Cell numbers and population percentages are shown. Representative of two independent experiments. Data are presented as mean values +/- SEM. Each data point represents the result from one mouse, and p values were calculated by two-sided student's t-test. Source data are provided as a Source Data file.

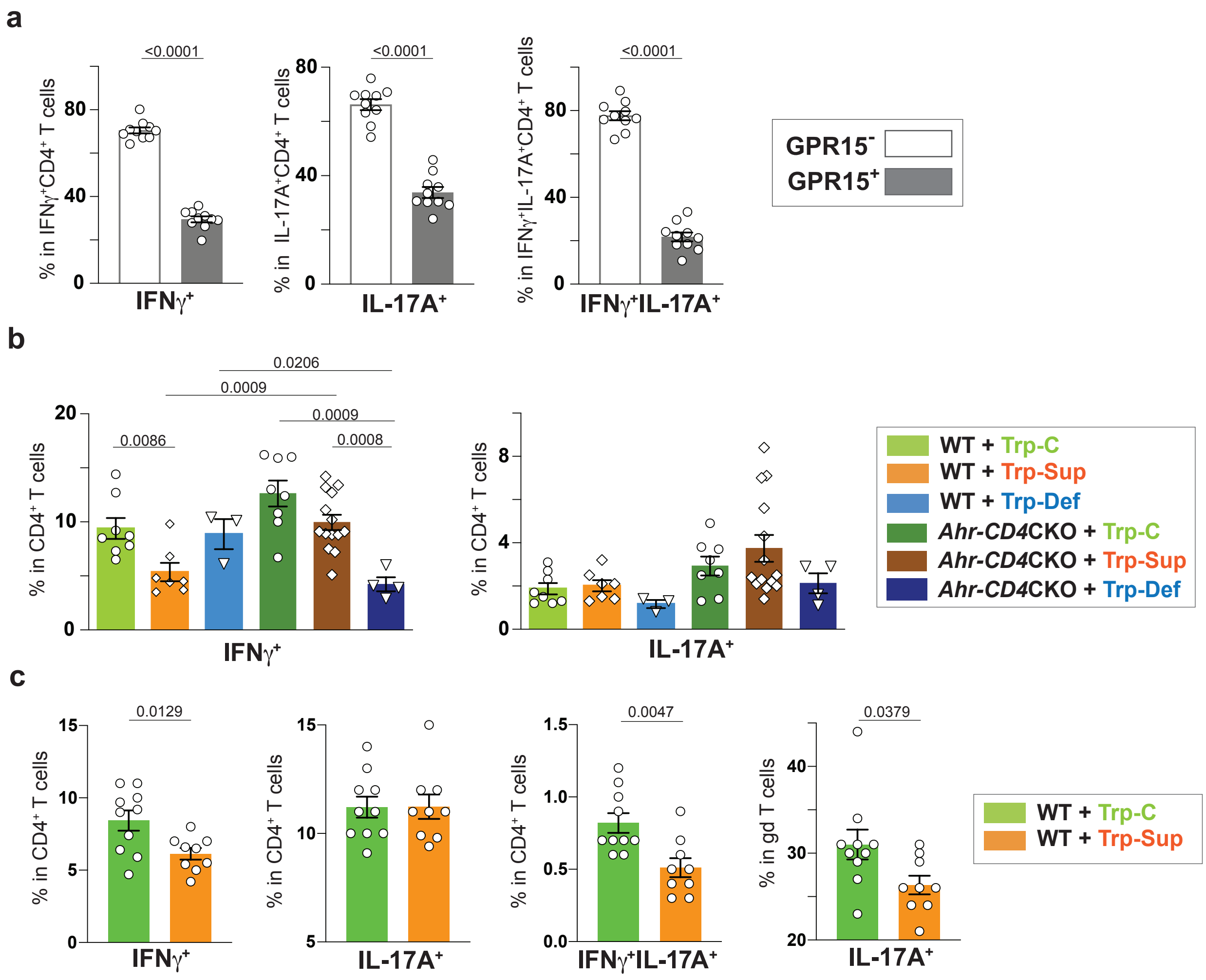

Supplementary Fig. 7 The decrease of IFNγ$^+$ cells and IFNγ$^+$IL-17A$^+$ accompanies dietary L-Trp-mediated increase of GPR15$^+$ Tregs in the large intestine.

a-c. Murine CD4$^+$ T cells from LILP were stimulated in the presence of GolgiStop, PMA, and Ionomycin for 3 hr and stained intracellularly for cytokines. **a.** 8-12-week-old wild-type C57BL/6 mice (n=10) at steady state with Taconic microbiota were used. The percentages of GPR15$^+$ and GPR15$^-$ cells among cytokine-producing cells were shown. Representative of two independent experiments. **b.** 8-14-week-old mice in C57BL/6 background with TJU microbiota 1 (SFB-free, Fig. 3b) were fed three different types of elementary diets *ad libitum* for three weeks as shown in Fig. 1c-d. Cells from WT (*Ahr*$^{(fl/fl)}$*Gpr15*$^{(gfp/+)}$*Foxp3*^{mrfp}) and *Ahr-CD4*CKO mice (*Cd4*^{Cre}*Ahr*$^{(fl/fl)}$*Gpr15*$^{(gfp/+)}$*Foxp3*^{mrfp}) were compared. Number of mice used: WT with Trp-C (n=8), Trp-Sup (n=7), or Trp-Def (n=3), *Ahr-CD4*CKO with Trp-C (n=8), Trp-Sup (n=14), or Trp-Def (n=4). Combined results of three independent experiments. **c.** 8-12-week-old wild-type C57BL/6 mice with Taconic microbiota were fed with Trp-C (n=10) or Trp-Sup (n=9) for 2 weeks and analyzed. Representative of two independent experiments. Data are presented as mean values +/- SEM (**a-c**). Each data point represents the result from one mouse, and p values were calculated by two-sided student's t-test (**a-c**). Source data are provided as a Source Data file (**a-c**).

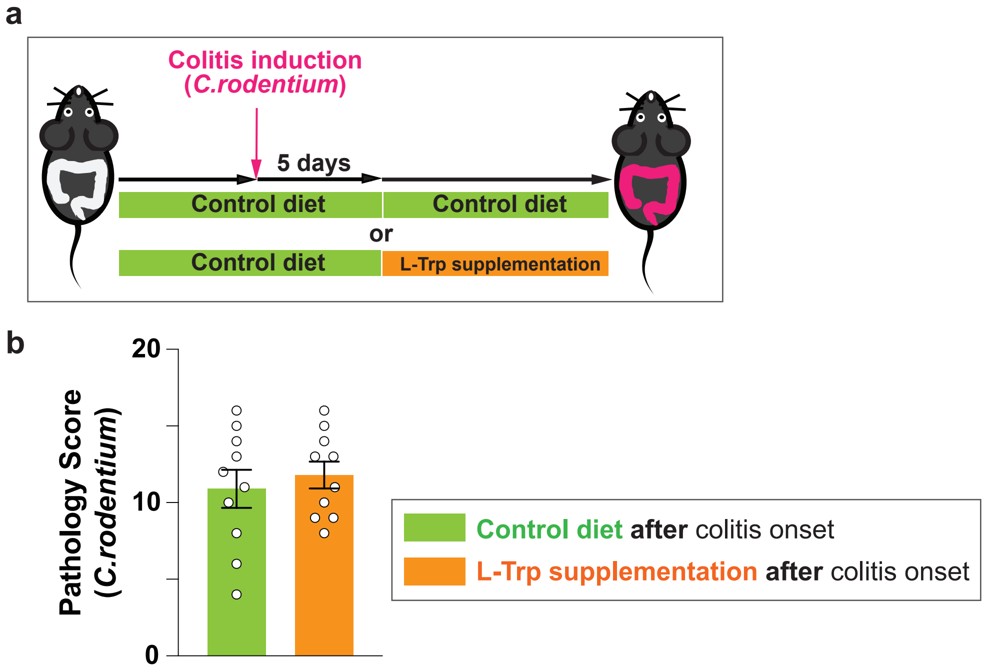

Supplementary Fig. 8 L-Trp supplementation after colitis onset does not reduce the severity of colitis. a. Experimental plan for the treatment of colitis**. b.** Pathology scores of the large intestine of 8-12-week-old wild-type C57BL/6 mice. Representative of two independent experiments. Number of mice used: WT with Control diet (n=10), WT with Trp-Sup (n=10). Data are presented as mean values +/- SEM. Each data point represents the result from one mouse. Source data are provided as a Source Data file (**b**).

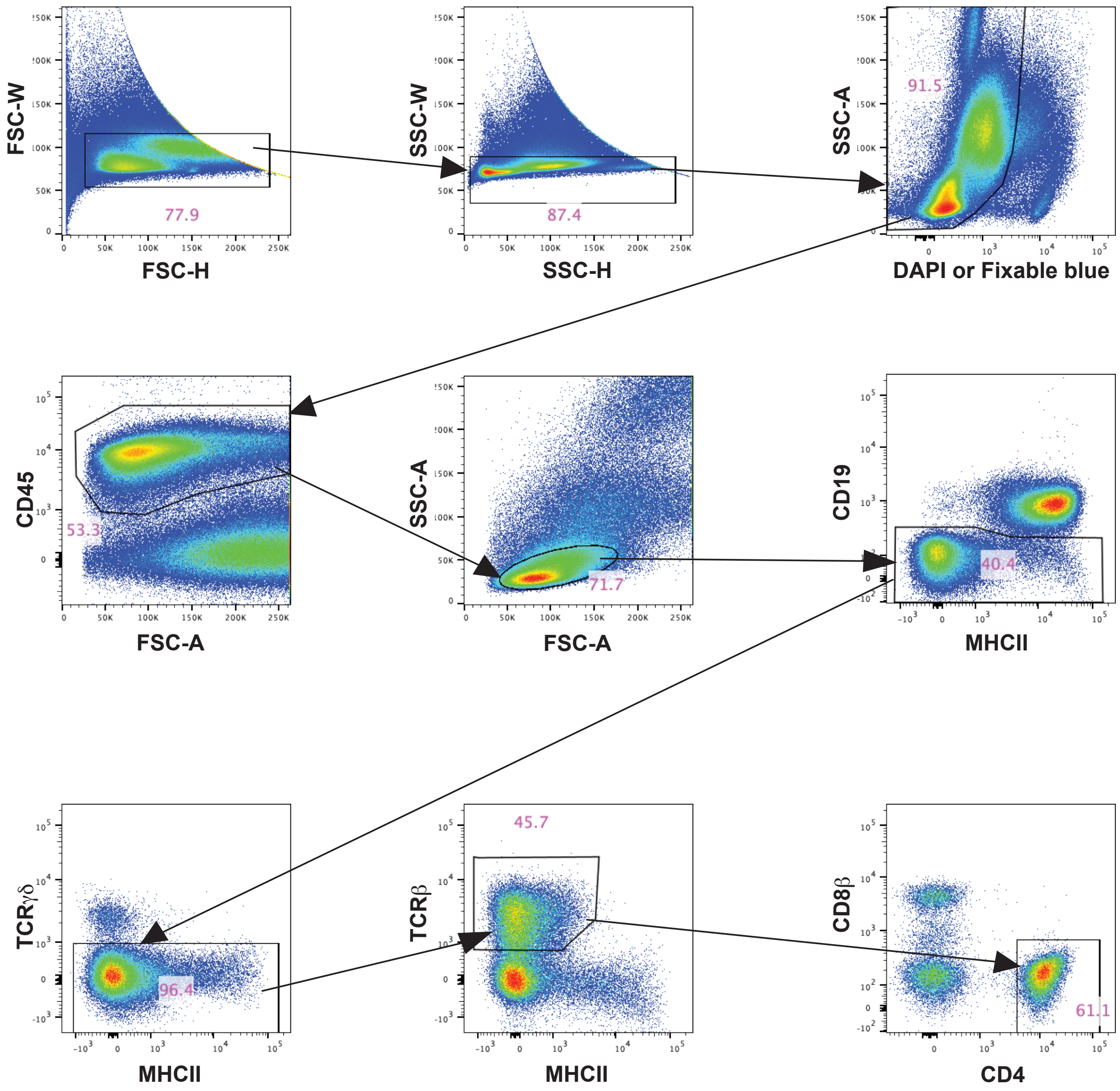

Supplementary Fig. 9 Gating strategy for CD4^{+} T cells. DAPI or Fixable blue were used to exclude dead cells from the analysis.

Supplementary Table 1. Composition of each elementary diet.

The total nitrogen intake of mice remained the same across different diets by adjusting the amount of non-essential amino acids.

Formula (g/kg)	Trp-C (TD.07788)	Trp-Sup (TD. 170745)	Trp-Def (TD. 08467)	Trp-Lo (TD. 200535)
L-Alanine	3.5	3.5	3.5	3.5
L-Arginine HCl	12.1	12.1	12.1	12.1
L-Asparagine	6.0	6.0	6.0	6.0
L-Aspartic Acid	3.5	3.5	3.5	3.5
L-Cystine	3.5	3.5	3.5	3.5
**L-Glutamic Acid**	**40.0**	**24.5**	**41.8**	**42.33**
Glycine	23.3	23.3	23.3	23.3
L-Histidine HCl, monohydrate	4.5	4.5	4.5	4.5
L-Isoleucine	8.2	8.2	8.2	8.2
L-Leucine	11.1	11.1	11.1	11.1
L-Lysine HCl	18.0	18.0	18.0	18.0
L-Methionine	8.6	8.6	8.6	8.6
L-Phenylalanine	7.5	7.5	7.5	7.5
L-Proline	3.5	3.5	3.5	3.5
L-Serine	3.5	3.5	3.5	3.5
L-Threonine	8.2	8.2	8.2	8.2
**L-Tryptophan**	**1.8**	**12.5**	**0**	**0.2**
L-Tyrosine	5.0	5.0	5.0	5.0
L-Valine	8.2	8.2	8.2	8.2
Sucrose	344.53	349.23	344.43	343.72
Corn Starch	150.0	150.0	150.0	150.0
Maltodextrin	150.0	150.0	150.0	150.0
Soybean Oil	80.0	80.0	80.0	80.0
Cellulose	30.0	30.0	30.0	30.0
Mineral Mix, AIN-93M-MX (94049)	35.0	35.0	35.0	35.0
Calcium Phosphate, monobasic, monohydrate	8.2	8.2	8.2	8.2
Vitamin Mix, AIN-93-VX (94047)	19.5	19.5	19.5	19.5
Choline Bitartrate	2.75	2.75	2.75	2.75
TBHQ, antioxidant	0.02	0.02	0.02	0.02
Food color		0.1	0.1	0.1
**Selected Nutrient Information (% by weight)**				
Protein	15.4	15.4	15.3	15.4
CHO	64.8	65.3	64.8	64.8
Fat	8	8	8	8

Supplementary Table 2. Chemical compounds used for in vitro screening in Fig. 4a

SOURCE	CHEMICAL NAMES	ABBREVIATION	CAS#	RANGE OF CONCENTRATION TESTED
Microbe-derived	**1**-Hydroxyphanazine	1-HP	528-71-2	1 nM-10 µM
	**1,4**-Dihydroxy-**2**-naphthoic acid	1,4-DH-2-NA	31519-22-9	1 nM-1 µM
	**2,8**-Quinolinediol		15450-76-7	1 nM-100 µM
	**3**-Methylindole	3M-indole	83-34-1	1 nM-100 µM
	Indole-**3**-acetaldehyde	I3-acetAld	20095-27-6	1 nM-10 µM
	Indole-**3**-acrylic acid	I3-acrylA	1204-06-4	1 nM-100 µM
	Indole-**3**-carboxaldehyde	I3-caAld	487-89-8	1 nM-100 µM
	**3**-Indolelactic acid	I3-LA	832-97-3	1 nM-1 mM
	Indole-**3**-propionic acid	I3-PA	830-96-6	1 nM-1 mM
	Indole-**3**-pyruvic acid	I3-PYA	392-12-1	1 nM-100 µM
	Indirubin		906748-38-7	1 nM-10 µM
	Indole		120-72-9	1 nM-100 µM
	Indoxyl sulfate	I3-S	2642-37-7	1 nM-100 µM
	Pyocyanin		85-66-5	1 nM-1 µM
Plant-derived	**3,3′**-diindolylmethane		1968-05-4	1 nM-10 µM
	Astaxanthin		472-61-7	1 nM-10 µM
	Benzothiazole		95-16-9	1 nM-100 µM
	Biochanin A		491-80-5	1 nM-10 µM
	Curcumin		458-37-7	1 nM-1 µM
	Diosmin		520-27-4	1 nM-100 µM
	Indole-**3**-acetic acid	I3-acetA	6505-45-9	1 nM-100 µM
	Indole-**3**-acetamide	I3-AM	879-37-8	1 nM-100 µM
	Indole-**3**-acetonitrile	I3-ACN	771-51-7	1 nM-100 µM
	Indole-**3**-carbinol	I3-CBL	700-06-1	1 nM-100 µM
	Indigo		482-89-3	1 nM-100 µM
	Myricetin		529-44-2	1 nM-100 µM
	Norisoboldine		23599-69-1	1 nM-10 µM
	Resveratrol		501-36-0	1 nM-10 µM
	Tryptamine		61-54-1	1 nM-10 µM
Host-derived	**3**-Hydroxyanthranilic acid	3H-anthranilic acid	548-93-6	1 nM-10 µM
	**3**-Hydroxy-DL-kynurenine	3H-Kyn	484-78-6	1 nM-100 µM
	**5**-hydroxy Indole-**3**-acetic Acid	5-HIAA	54-16-0	10 nM-100 µM
	**5**-Hydroxy-L-Tryptophan	5-HTP	4350-09-8	10 nM-100 µM
	**5**S,**6**R-dihydroxy-**7**E,**9**E,**11**Z,**14**Z-eicosatetraenoic acid	5(S),6(R)-DiHETE	82948-88-7	1 nM-100 µM
	Anthranilic acid		118-92-3	1 nM-100 µM
	Bilirubin		635-65-4	1 nM-10 µM

	Biliverdin			1 nM-100 µM
	Cinnabarinic acid		606-59-7	1 nM-100 µM
	**6**-Fomylindolo(**3**,**2**-b)carbazole	FICZ	172922-91-7	
	**2**-(**1**H-Indol-**3**-ylcarbonyl)-**4**-thiazolecarboxylic acid methyl ester	ITE	448906-42-1	1 nM-100 µM
	Kynurenic acid		492-27-3	1 nM-100 µM
	L-Kynurenine		2922-83-0	1 nM-100 µM
	Melatonin			10 nM-100 µM
	Picolinic acid			
	Prostaglandin G**2**		51982-36-6	1 nM-10 µM
	Quinolinic acid		89-00-9	1 nM-100 µM
	Serotonin			1 nM-100 µM
	Xanthurenic acid		59-00-7	1 nM-100 µM
Anthropogenic	**1**,**2**-Naphthoquinone		524-42-5	1 nM-1 µM
	**1**,**4**-Naphthoquinone		130-15-4	1 nM-1 µM
	**10**-chloro-**7**H-benzimidazo[**2**,**1**-a]benzo[de]isoquinolin-**7**-one	10-CL-BBQ	23982-76-5	1 nM-10 nM
	**7**,**12**-Dimethylbenz[a]anthracene	7,12-DMBA	57-97-6	1 nM-10 µM
	Alpha-naphthoflavone	Alpha-NF	604-59-1	1 nM-10 µM
	(Z)-**2**-(**3**,**4**-dichlorophenyl)-**3**-(1H-pyrrol-**2**-yl)acrylonitrile	ANI-7	931417-26-4	1 nM-10 µM
	Benzo[a]pyrene	BaP	50-32-8	1 nM-10 µM
	Beta-napthoflavone	Beta-NF	6051-87-2	1 nM-100 µM
	Leuflunomide		75706-12-6	1 nM-10 µM
	Mesalamine		89-57-6	1 nM-100 µM
	**1**-allyl-**3**-(**3**,**4**-dimethoxyphenyl)-**7**-(trifluoromethyl)-**1**H-indazole	SGA360	680611-86-3	1 nM-10 µM
	**7**-Oxo-**7***H*-benzimidazo[**2**,**1**-*a*]benz[de]isoquinoline-**3**-carboxylic acid	STO-609		1 nM-10 µM
	**2**,**3**,**7**,**8**-tetrachlorodibenzo-*p*-dioxin	TCDD	1746-01-6	1 nM-100 nM
	**6**,**2**,**4**-Trimethoxyflavone	TMF	720675-90-1	1 nM-10 µM
