# Supplementary material for: Dietary L-Tryptophan consumption determines the number of colonic regulatory T cells and susceptibility to colitis via GPR15
Source: Nat Commun. 2023 Nov 14;14:7363. doi: 10.1038/s41467-023-43211-4 (PMC10645889; doi:10.1038/s41467-023-43211-4)
Supplement: Supplementary file 3 — Reporting Summary [file 41467_2023_43211_MOESM3_ESM.pdf]

nature portfolio

Corresponding author(s): Sangwon V. Kim

Last updated by author(s): Sep 29, 2023

Reporting Summary

Nature Portfolio wishes to improve the reproducibility of the work that we publish. This form provides structure for consistency and transparency in reporting. For further information on Nature Portfolio policies, see our Editorial Policies and the Editorial Policy Checklist.

Statistics

For all statistical analyses, confirm that the following items are present in the figure legend, table legend, main text, or Methods section.

n/a	Confirmed	
☐	☒	The exact sample size (*n*) for each experimental group/condition, given as a discrete number and unit of measurement
☐	☒	A statement on whether measurements were taken from distinct samples or whether the same sample was measured repeatedly
☐	☒	The statistical test(s) used AND whether they are one- or two-sided<br>*Only common tests should be described solely by name; describe more complex techniques in the Methods section.*
☒	☐	A description of all covariates tested
☒	☐	A description of any assumptions or corrections, such as tests of normality and adjustment for multiple comparisons
☐	☒	A full description of the statistical parameters including central tendency (e.g. means) or other basic estimates (e.g. regression coefficient) AND variation (e.g. standard deviation) or associated estimates of uncertainty (e.g. confidence intervals)
☐	☒	For null hypothesis testing, the test statistic (e.g. *F*, *t*, *r*) with confidence intervals, effect sizes, degrees of freedom and *P* value noted<br>*Give P values as exact values whenever suitable.*
☒	☐	For Bayesian analysis, information on the choice of priors and Markov chain Monte Carlo settings
☒	☐	For hierarchical and complex designs, identification of the appropriate level for tests and full reporting of outcomes
☒	☐	Estimates of effect sizes (e.g. Cohen's *d*, Pearson's *r*), indicating how they were calculated

Our web collection on statistics for biologists contains articles on many of the points above.

Software and code

Policy information about availability of computer code

Data collection	FACSDiva software (Version 8; BD) was used to collect data from flow cytometers.
Data analysis	FlowJo software (Version 10; BD) was used for the analysis of the flow cytometry data. Prism (Version 10; GraphPad) software was used for statistical analysis.

For manuscripts utilizing custom algorithms or software that are central to the research but not yet described in published literature, software must be made available to editors and reviewers. We strongly encourage code deposition in a community repository (e.g. GitHub). See the Nature Portfolio guidelines for submitting code & software for further information.

Data

Policy information about availability of data

All manuscripts must include a data availability statement. This statement should provide the following information, where applicable:

- Accession codes, unique identifiers, or web links for publicly available datasets
- A description of any restrictions on data availability
- For clinical datasets or third party data, please ensure that the statement adheres to our policy

Source data generated in this study are provided in the Supplementary Information/Source Data file. The raw flow cytometry data generated in this study has been deposited in the flow repository database at http://flowrepository.org under accession codes as follows: FR-FCM-Z6S8 for Figures 1b, 1c, and 3a (TJU); FR-FCM-Z6S9 for Figure 1d; FR-FCM-Z6SA for Figures 1e, 3c (JAX), 6f (mouse), and 6g (mouse): FR-FCM-Z6SB for Figures 2a, 2b, and 2c; FR-FCM-Z6SC for Figures 2e and 2f; FR-FCM-Z6S5 for Figure 3b (TJU2); FR-FCM-Z6S4 for Figure 3c (TAC); FR-FCM-Z6SD for Figure 3d; FR-FCM-Z6SE for Figure 3e; FR-FCM-Z6SF for Figure 3f; FR-FCM-Z6T6,

FR-FCM-Z6T7, FR-FCM-Z6T9, and FR-FCM-Z6TA for Figure 4a; FR-FCM-Z6SL for Figure 4b; FR-FCM-Z6TC for Figure 5a; FR-FCM-Z6SX for Figures 5c and S6; FR-FCM-Z6SP for Figures 6f and 6g; FR-FCM-Z6SQ for Figure S1; FR-FCM-Z6SR for Figure S2; FR-FCM-Z6SS for Figure S3; FR-FCM-Z6ST for Figure S4a; FR-FCM-Z6SU for Figure S4b; FR-FCM-Z6SV for Figure S5; FR-FCM-Z6TZ for Figures S7a and S7c; FR-FCM-Z6TY for Figure S7b.

Research involving human participants, their data, or biological material

Policy information about studies with human participants or human data. See also policy information about sex, gender (identity/presentation), and sexual orientation and race, ethnicity and racism.

Reporting on sex and gender	Before being received for analysis, all human specimens were de-identified. As a result, we lack information on sex- and gender.
Reporting on race, ethnicity, or other socially relevant groupings	Before being received for analysis, all human specimens were de-identified. As a result, we lack information on race, ethnicity, or other socially relevant groupings.
Population characteristics	Before being received for analysis, all human specimens were de-identified. As a result, we lack information on the population's characteristics. Some specimens were taken from resections during surgeries for colon cancer patients; in those circumstances, normal resections free of cancer tissues were used for our investigation.
Recruitment	Volunteering participants included adult subjects undergoing colonoscopy at Weill Cornell hospital or undergoing colorectal resection at Thomas Jefferson University Hospital without restrictions based on sex/gender, race/ethnicity, or age.
Ethics oversight	Weill Cornell Medicine IRB approved protocol (1103011578); Thomas Jefferson University IRB approved protocol (18D.495)

Note that full information on the approval of the study protocol must also be provided in the manuscript.

Field-specific reporting

Please select the one below that is the best fit for your research. If you are not sure, read the appropriate sections before making your selection.

- [x] Life sciences
- [ ] Behavioural & social sciences
- [ ] Ecological, evolutionary & environmental sciences

For a reference copy of the document with all sections, see nature.com/documents/nr-reporting-summary-flat.pdf

Life sciences study design

All studies must disclose on these points even when the disclosure is negative.

Sample size	In vivo mouse experiments used the number of animals in accordance with the Guidelines of the Institutional Animal Care and Use Committee (IACUC). Sample size for mouse experiments was determined by feasibility and the magnitude of the interventions impact.
Data exclusions	Data from mice that succumbed to colitis were excluded from data analysis
Replication	Replication numbers of each experiment are written in the manuscript. Repeated at least three times: Figures 1d,1e, 4b, S1, and S5; repeated at least twice: Figures 1e, 2a, 2e, 2f, 3b, 3c , 3e, 4c, 5a, S7a, and S7b; combined results of two or more repeated experiments: Figures 2b, 2c, 3f, 4a, 5c, 6b, 6c, 6d, 6e, 6f, S3, S4, S6, S7b and S8b.
Randomization	Mice were either littermates or cohoused for at least two week prior to the experiments.
Blinding	For mouse colitis experiments, representative sections with Hematoxylin/Eosin staining were evaluated in a double-blind manner by two gastrointestinal pathologists. For flow cytometry analysis, the same gating strategies were applied blindly to all FCS files for each analysis regardless of experimental groups.

Reporting for specific materials, systems and methods

We require information from authors about some types of materials, experimental systems and methods used in many studies. Here, indicate whether each material, system or method listed is relevant to your study. If you are not sure if a list item applies to your research, read the appropriate section before selecting a response.

Materials & experimental systems

n/a	Involved in the study	
	☒	Antibodies
☒		Eukaryotic cell lines
☒		Palaeontology and archaeology
	☒	Animals and other organisms
☒		Clinical data
☒		Dual use research of concern
☒		Plants

Methods

n/a	Involved in the study	
☒		ChIP-seq
	☒	Flow cytometry
☒		MRI-based neuroimaging

Antibodies

Antibodies used	FITC-mCD19 (1:400 dilution; Clone: 1D3/CD19, Biolegend 152404), PerCP-Cy5.5-mTCRgd (1:400 dilution; Clone: GL3, Biolegend 118118), PE-mFOXP3 (1:100 dilution; Clone: FJK-16s, Thermofisher 12-5773-82), APC-mRORgt (1:100 dilution; Clone: B2D, Thermofisher 17-6981-82), Alexa 488-mHELIOS (1:100 dilution; Clone: 22F6, Biolegend 137223), Biotin-mGPR15 (1:20 dilution; Clone: S15042I, Biolegend 154602), Alexa 700-mI-A/I-E (1:800 dilution; Cone: M5/114.15.2, Biolegend 107622), APCFire-mTCRb (1:400 dilution; Clone: H57-597, Biolegend 109246), Pacific blue or BV421-mCD8b (1:1000 dilution; Clone: 53-5.8 or YTS156.7.7, Biolegend 140414 or 126629), BV605-mNK1.1 (1:400 dilution; Clone: PK136, Biolegend 108753), BV650-mCD45 (1:4000 dilution; Clone: 30-F11, Biolegend 103151), BV785-mCD4 (1:400 dilution; Clone: RM4-5, Biolegend 100552), mCD16/32 (1:200 dilution; Clone: 2.4G2, Tonbo Bioscience 30-0161-U500), PE-mCD45.2 (1:400 dilution; Clone: 104, Biolegend 109808), Pacific-blue-mCD45.1 (1:400 dilution; Clone: A20, Biolegend 110722), APC-mTCRVb6 (1:400 dilution; Clone: RR4-7, Biolegend 140006), mCD3e (Clone: 145-2c11, Biolegend 100340), mCD28 (Clone: 37.51, Biolegend 102116), hamster IgG (polyclonal, MPbio 855398 or Sigma SAB3700488), PE-IFNg (1:100 dilution; Clone: XMG1.2, Biolegend 505808), APC-IL17A (1:100 dilution; Clone: eBio17B7, Thermofisher 17-7177-81), FITC-hCD45RA (1:33 dilution; Clone: HI100, Biolegend 304148), PE-hCD25 (1:20 dilution; Clone: BC96, Biolegend 302606), PECy7-hCD3e (1:33 dilution; Clone:UCHT1, Biolegend 300420), PerCPCy5.5-HLA-DR (1:33 dilution; Clone: LN3, Biolegend 327020), APC-hGPR15 (1:20 dilution; Clone: SA302A10, Biolegend or Clone 3679902, R&D), Alexa780-hCD4 (1:33 dilution; Clone: RPA-T4, Biolegend 300518), PE-hFOXP3 (1:100 dilution; Clone: 236A/E7, Thermofisher 12-4777-42).
Validation	Flow cytometry antibodies validated by manufacturer (Biolegend, Thermofisher, R&D systems, Tonbo bio, MPBio, and Sigma). IFN-g and IL-17 antibodies have been reported for use in intracellular staining. RORgt, FOXP3, HELIOS antibodies have been reported for use in intra-nuclear staining followed by flow cytometry analysis.

Animals and other research organisms

Policy information about studies involving animals; ARRIVE guidelines recommended for reporting animal research, and Sex and Gender in Research

Laboratory animals	C57BL/6 mice were obtained from either Jackson Laboratory or Taconic Biosciences. Gpr15gfp mice were described previously (references included in the manuscript). Ahr(fl/fl) mice, CD4cre mice, Cd45(1/1) mice, and Thy1(1/1) mice were obtained from Jackson Laboratory. Foxp3mrfp mice, Ido2(n/n), Ido1/2(n/n), Tdo2(n/n), Tph1(n/n), and HH7-2 TCR transgenic mice were described previously (references included in the manuscript). GPR15gfpFOXP3mrfp mice were generated by crossing Gpr15gfp mice and Foxp3mrfp mice. Ahr(n/n) mice were generated by crossing Ahr(fl/fl) mice with EIIacre mice from Jackson Laboratory. Gpr15TFdeletion, Gpr15DREmut1 mice were generated by improved-Genome editing via Oviductal Nucleic Acids Delivery (iGONAD) method at Thomas Jefferson University. 8-14 week old mice were used for the study. Both males and female mice were used. Mice were housed at 12 hours/12 hours dark/ light cycles with temperature at 70 +/- 2 degrees and humidity of 30-70 % in specific-pathogen-free conditions.
Wild animals	None.
Reporting on sex	Sex was not considered in this study design since our preliminary experiments with diets of various L-Trp contents showed no differences based on sex.
Field-collected samples	None
Ethics oversight	IACUC at Thomas Jefferson University

Note that full information on the approval of the study protocol must also be provided in the manuscript.

Flow Cytometry

Plots

Confirm that:

- [x] The axis labels state the marker and fluorochrome used (e.g. CD4-FITC).
- [x] The axis scales are clearly visible. Include numbers along axes only for bottom left plot of group (a 'group' is an analysis of identical markers).
- [x] All plots are contour plots with outliers or pseudocolor plots.
- [x] A numerical value for number of cells or percentage (with statistics) is provided.

Methodology

Sample preparation	For the large intestine, cecum and colon were collected, cut open, and cut into 4 pieces without cecal patches. They were washed with ice-cold PBS by hand-shaking and treated with 1mM DTT/PBS for 10 min, followed by two consecutive treatments with 30mM EDTA/PBS at room temperature in the shaker (100 rpm/min). Tissue pieces were washed with PBS once, cut into smaller pieces of about 0.5 mm x 0.5 mm, and incubated in the digestion solution (collagenase 8 [Sigma], dispase [Worthington], DNase I [Sigma] in RPMI with 10% fetal bovine serum) at 37°C with occasional shaking for 1-1.5hrs. Digested tissues were filtered through 100mm filter and cells were recovered at the interface between 40% and 80% percoll (GE healthcare) after spinning at 850g for 20 min. For the small intestine, Peyer's patches were removed, cut open, and cut into 8-12 pieces. Subsequently, samples were washed with ice-cold PBS once and treated with 5mM EDTA/PBS for 10 min at the shaker (100 rpm/min) set at 37°C. The rest of the steps for cell isolation after EDTA/PBS treatment is the same as described above for the large intestine. Human samples were processed in the same procedure as described for the mouse large intestine except that collagenase D (Roche) was used instead of collagenase 8 (Sigma) in the digestion solution.
Instrument	BD Fortessa analyzer, BD Aria II sorter
Software	FACSDiva software (Version 8; BD) was used to collect data from flow cytometers. FlowJo software (Version 10) was used for the analysis.
Cell population abundance	The purity of sorted cells was more than 99%.
Gating strategy	The following gating strategy was used to identify single, live lymphocytes: FSC-H/FSC-W, SSC-H/SSC-W, DAPI/SSC-A, FSC-A/SSC-A

- [x] Tick this box to confirm that a figure exemplifying the gating strategy is provided in the Supplementary Information.
